# Benchmarking reinforcement learning algorithms for autonomous mechanical thrombectomy

**DOI:** 10.1007/s11548-025-03360-x

**Published:** 2025-04-29

**Authors:** Farhana Moosa, Harry Robertshaw, Lennart Karstensen, Thomas C. Booth, Alejandro Granados

**Affiliations:** 1 School of Biomedical Engineering and Imaging Sciences, Kings College London, London, UK; 2 AIBE, Friedrich-Alexander University Erlangen-Nürnberg, Erlangen, Germany; 3 Department of Neuroradiology, Kings College Hospital, London, UK

**Keywords:** Reinforcement learning, Mechanical thrombectomy, Machine learning, Artificial intelligence, Autonomous navigation, Endovascular intervention

## Abstract

**Purpose:**

Mechanical thrombectomy (MT) is the gold standard for treating acute ischemic stroke. However, challenges such as operator radiation exposure, reliance on operator experience, and limited treatment access remain. Although autonomous robotics could mitigate some of these limitations, current research lacks benchmarking of reinforcement learning (RL) algorithms for MT. This study aims to evaluate the performance of Deep Deterministic Policy Gradient, Twin Delayed Deep Deterministic Policy Gradient, Soft Actor-Critic, and Proximal Policy Optimization for MT.

**Methods:**

Simulated endovascular interventions based on the open-source stEVE platform were employed to train and evaluate RL algorithms. We simulated navigation of a guidewire from the descending aorta to the supra-aortic arteries, a key phase in MT. The impact of tuning hyperparameters, such as learning rate and network size, was explored. Optimized hyperparameters were used for assessment on an MT benchmark.

**Results:**

Before tuning, Deep Deterministic Policy Gradient had the highest success rate at 80% with a procedure time of 6.87 s when navigating to the supra-aortic arteries. After tuning, Proximal Policy Optimization achieved the highest success rate at 84% with a procedure time of 5.08 s. On the MT benchmark, Twin Delayed Deep Deterministic Policy Gradient recorded the highest success rate at 68% with a procedure time of 214.05 s.

**Conclusion:**

This work advances autonomous endovascular navigation by establishing a benchmark for MT. The results emphasize the importance of hyperparameter tuning on the performance of RL algorithms. Future research should extend this benchmark to identify the most effective RL algorithm.

**Supplementary Information:**

The online version contains supplementary material available at 10.1007/s11548-025-03360-x.

## Introduction

Stroke remains a leading cause of mortality and long-term disability worldwide, affecting over 12.2 million people each year [3]. Mechanical thrombectomy (MT) is recognized as the gold standard for treating acute ischemic strokes caused by large vessel occlusions [16], and it has been shown to improve patient outcomes compared to standard treatment alone [22]. The procedure, supported by fluoroscopic imaging, involves a clinician navigating a guidewire and catheter from the common femoral artery, through the common iliac artery, aorta, and internal carotid arteries (ICAs), to a target site in the brain. A device at the catheter’s distal end, such as a stent retriever or aspiration system, is then deployed to capture and remove the thrombus, restoring blood flow and reducing the severity of disabilities caused by strokes [21].

Despite its efficacy, the success of MT relies on timely treatment and operator experience. Increased operator experience is linked to shorter procedure durations and better reperfusion outcomes [26], while delays from symptom onset to treatment correlate with higher disability levels [24]. Yet, of the 10% of patients qualifying for MT, only 3.1% are eligible due to limited access and shortage of expertise [12, 17]. Robotic platforms with teleoperated controller-operator architectures have been developed to address these challenges. However, these systems require skills not typical in clinical practice and lack haptic feedback, complicating training [7]. Recent advancements in machine learning (ML) have led to autonomous solutions for robotic procedures, which could shift clinicians to a supervisory role and expand access to MT [9, 23].

In recent literature, we have witnessed a range of approaches to address challenges in endovascular surgery using reinforcement learning (RL) [20]. However, considerable variability in study designs, evaluation metrics, and testing environments poses challenges for the quantitative comparison of RL approaches [20]. For example, Meng et al. and Ritter et al. investigated autonomous navigation from the descending aorta to the supra-aortic arteries through *in silico* experimentation [13, 19]. Meng et al. compared autonomous versus manual navigation of a catheter and guidewire using the Asynchronous Advantage Actor-Critic (A3C) algorithm, focusing on metrics like contact force and procedure time. In contrast, Ritter et al. examined guidewire navigation using different reward functions for the Soft Actor-Critic (SAC) algorithm, evaluating metrics such as success rate and path length ratio. Despite similar objectives, differences in simulation environments, approaches, and metrics hinder direct comparison of their results. Similarly, Behr et al. conducted an *in vitro* study on autonomous guidewire navigation through a vascular phantom with bifurcation and trifurcation, utilizing Deep-Q-Network (DQN) and Deep Deterministic Policy Gradient (DDPG) algorithms enhanced by Hindsight Experience Replay (HER) and Human Demonstration (HD), measuring success rate as a key performance metric [1]. Cho et al. combined DDPG with Behavior Cloning on a simplified vascular model, focusing on procedure time [2]. Without proper benchmarking, the variability in study designs and RL implementations across these works continues to challenge the identification of the most effective algorithms for future research.

To address these issues, Karstensen et al. and Jianu et al. introduced open-source simulation environments for endovascular interventions, named stEVE and CathSim, respectively, [8, 10]. stEVE features three benchmark interventions: Two are designed for guidewire navigation from the descending aorta to the supra-aortic arteries, which have proven *in vitro* benefits, generalization capabilities, and adaptability to patient-specific variations; the third is the first dedicated MT benchmark [9, 10]. Baseline results for a custom SAC implementation were established for each of these standards [10]. Conversely, CathSim focuses on navigation tasks from the ascending aorta through two aortic arch vasculatures, with baseline results provided for Proximal Policy Optimization (PPO) and SAC algorithms from the Stable Baselines3 (SB3) library [8, 18]. Unlike stEVE, CathSim lacks *in vitro* validation and generalization capabilities. Despite this progress, several challenges remain. First, while these studies offer valuable insights into the performance of RL algorithms, they fall short in comparing algorithmic performance differences comprehensively. Second, the impact of hyperparameter tuning is not sufficiently addressed. Third, while stEVE provides a wider range of evaluation environments, it lacks baseline results with readily available RL algorithms. Consequently, we chose stEVE over CathSim for its specific MT benchmark and established *in vitro* validation and generalization capabilities, critical for assessing the efficacy of RL algorithms under more realistic and varied clinical scenarios.

The aim of this study was to evaluate and compare four model-free RL algorithms from the SB3 library: DDPG, Twin Delayed DDPG (TD3), SAC, and PPO, using stEVE in the context of MT. Initially, we assessed these algorithms in a simulation task designed to navigate a guidewire from the descending aorta to the supra-aortic arteries, a key phase in MT. Next, we explored the impact of tuning hyperparameters, such as learning rate and network size, on algorithm performance. Finally, the optimized hyperparameters were used to assess the algorithms on an MT benchmark, navigating a guidewire and catheter from the common iliac artery to a target in the ICAs.

## Methods

### Navigation tasks and simulation environments

In this work, we utilized the ArchVariety and DualDeviceNav environments from stEVE to facilitate evaluations of simple and complex navigation tasks, respectively, thus providing insights into varying levels of procedural difficulty.

**Fig. 1:**
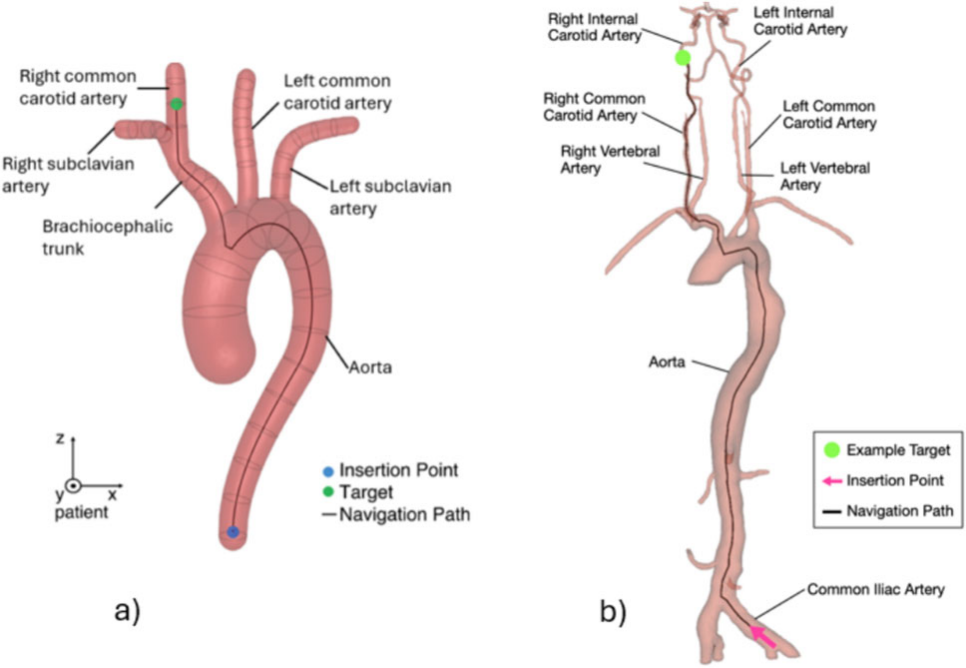
**a** ArchVariety environment with a Type I aortic arch, labeled anatomy, insertion point, and exemplary navigation path to the right common carotid artery. **b** DualDeviceNav setup illustrating the simulation, insertion point, exemplary navigation path to the right ICA, and labeled anatomy

The navigation task facilitated by ArchVariety involves maneuvering a guidewire through a three-dimensional synthetic aortic arch. Starting from the descending aorta and extending to the supra-aortic arteries, the objective is to navigate the guidewire from an insertion point at the model’s caudal end to a target randomly sampled along the centerlines of the supra-aortic arteries [10]. The target branches considered here included the subclavian arteries, carotid arteries, and brachiocephalic trunk. A Type I aortic arch was selected due to its predominance in clinical scenarios [15]. Figure 1(a) displays the ArchVariety environment.

The navigation task in DualDeviceNav builds on ArchVariety. Unlike the standard DualDeviceNav setup, target locations considered here are restricted to the left and right ICAs [21]. The task involves navigating a catheter and guidewire from an insertion point in the common iliac artery through the aorta to targets randomly sampled from 20 centerline points within the right or left ICAs. The simulation environment with labeled anatomy is illustrated in Fig. 1(b).

### RL algorithms, training procedure, and evaluation

This study employs implementations of DDPG, TD3, SAC, and PPO from SB3. DDPG, TD3, and SAC are all off-policy algorithms. DDPG utilizes a deterministic policy to maximize Q-values, effective for environments with continuous action spaces [11]. TD3 builds on it by addressing overestimation bias with twin Q-networks and delayed policy updates, enhancing training stability [4]. SAC employs an entropy maximization strategy to encourage exploration and improve policy robustness [5]. Contrastingly, PPO, an on-policy algorithm, uses a clipping mechanism in its objective function to prevent large updates, ensuring stable, reliable policy improvement [25].

The inputs for all control algorithms consist of the instrument’s current position, its two preceding positions, the target position, and the last action executed. These elements collectively form the observation set. The current position is specified by tracking coordinates at three points on the instrument’s tip, denoted as $(x', z')_i$ for $i = \{1, 2, 3\}$, where $(x', z')_1$ aligns with the tip of the instrument. These points are uniformly spaced, with a 2-mm gap between each. The target position is represented as $(x', z')$, indicating the current goal. Actions, which are the outputs of the policy network, consist of the instrument’s rotational and translational speeds. These speeds are limited to 35 mm/s and 3.14 rad/s, respectively [10]. Furthermore, the tracking coordinate system here is a rotated projection of the patient coordinate system, as explained in [9], designed to maximize visibility in fluoroscopic images and provide feedback equivalent to two-dimensional tracking typically used in clinical procedures.

The neural network architecture used in this study is based on the SB3 framework. These networks utilize a feed-forward structure with two fully connected hidden layers. Table S1 in Online Resource 1 summarizes the default hyperparameters used for each algorithm. Gaussian action space noise with a mean of 0 and a standard deviation of $\sigma = 0.1$ was introduced to the deterministic policy frameworks of DDPG and TD3 to facilitate exploration, based on values documented in the literature [6, 14].

The reward function employed is adopted from the work of [9] and utilizes a dense reward structure, defined as follows:

1 $$\begin{aligned} R = -0.005 - 0.001 \times \Delta \textit{pathlength} + {\left\{ \begin{array}{ll} 1.0 &  \text {if target is reached} \\ 0 &  \text {otherwise} \end{array}\right. } \end{aligned}$$

where *pathlength* refers to the distance between the tip of the instrument and the target, measured along the centerlines of the arteries. The term $\Delta pathlength$ represents changes in pathlength from the previous to the current timestep.

The training procedure involves navigating the instrument from an initial starting point to a designated target over $1 \times 10^7$ exploration steps. Each navigation task, defined as an episode, is considered complete when the target is reached within a 5-mm tolerance or when a truncation timeout occurs, either due to reaching the maximum exploration steps (200 for ArchVariety and 500 for DualDeviceNav) or when the instrument reaches the end of a vessel.

To track training progress, evaluations were conducted every $2.5 \times 10^5$ exploration steps for 100 episodes. Key metrics recorded during each evaluation step, success rate, procedural time, and path ratio, were chosen for their clinical relevance and frequent use in related research, facilitating easier comparisons. Comparison between algorithms was made based on the highest success rate achieved for each algorithm, along with the corresponding path ratio and procedure time recorded at the same evaluation step. *Success Rate* refers to the percentage of evaluation episodes in which the control algorithm successfully reached the target. *Path Ratio* represents the total distance traveled divided by the initial distance to the target in unsuccessful episodes. *Procedure Time* measures the duration from the start of navigation to reaching the target in successful episodes. Training and evaluation were conducted using an NVIDIA DGX A100 node equipped with 8 GPUs. Typical training times were 120 h (DDPG, TD3, SAC) and 84 h (PPO) for ArchVariety and 240 h (DDPG, TD3, SAC) and 192 h (PPO) for DualDeviceNav.

### Experiments

#### Default hyperparameters in ArchVariety environment

In this experiment, algorithms were trained in ArchVariety. For each navigation task, artery geometries were randomized using a number generator with seeds from 0 to $2^{31}$. A finite set of 100 seeds was used during the evaluation steps to ensure consistent comparisons across all algorithms, and the initial model weights seed was fixed at 42. The objective was to assess algorithm performance under default hyperparameter settings.

#### Tuned hyperparameters in ArchVariety environment

This experiment focused on tuning the neural network size and learning rate for each algorithm. The performance of DDPG, TD3, SAC, and PPO was evaluated using both small and large network configurations, with sizes of (64, 64) and (400, 300), respectively, to assess how increased network capacity affects each algorithm’s performance. Success rates were monitored throughout training to identify the optimal network configuration for each algorithm. Once the optimal network size was determined, the default learning rate was used as a baseline, followed by the exploration of two additional values selected through a preliminary grid search (see Table S2 in Online Resource 1). All other hyperparameters remained at their default settings, with the training and evaluation environment consistent with the first experiment. The goal was to evaluate the impact of network size and learning rate adjustments on algorithm performance.

#### Tuned hyperparameters in DualDeviceNav environment

In this experiment, algorithms were trained in DualDeviceNav to assess learning in complex environments and evaluate susceptibility to overfitting. Success rates were monitored across all evaluation steps to establish a baseline performance.

## Results

The highest success rate achieved during the evaluation steps of each experiment, along with the corresponding path ratio and procedure time, is presented in Table 1. Videos showing the navigation process are provided in Online Resource 2.

### Default hyperparameters in ArchVariety environment

**Fig. 2:**
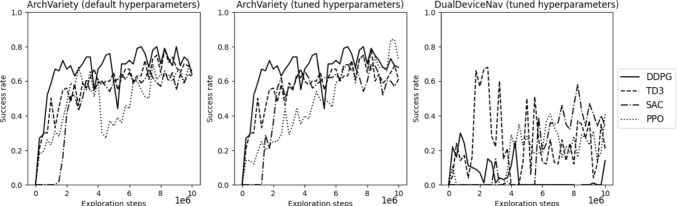
Comparison of success rates during training: (a) default hyperparameters in ArchVariety, (b) tuned hyperparameters in ArchVariety, and (c) tuned hyperparameters in DualDeviceNav. Additionally, training curves for ArchVariety and DualDeviceNav are provided in Online Resource 1

The initial assessment in ArchVariety highlighted that DDPG and TD3 were the most effective algorithms, achieving maximum success rates of 80% and 79%, respectively, with DDPG also recording a notably faster average procedure time of 6.87 s. In contrast, SAC had the lowest maximum success rate among the tested algorithms at 66%, while PPO was the fastest with a procedure time of 5.5 s. Figure 2(a) illustrates the success rates of the algorithms during training with default hyperparameters.

### Tuned hyperparameters in ArchVariety environment

**Fig. 3:**
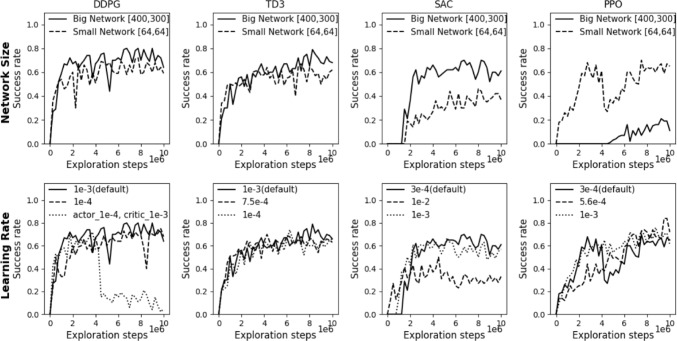
Comparison of network size (top row) and learning rate (bottom row) on success rate during training in ArchVariety for four RL algorithms

Further investigations explored how network size and learning rate changes impact each algorithm’s performance. Larger networks generally benefited off-policy algorithms, with SAC showing the most significant improvements in success rates, as shown in Fig. 3. Conversely, PPO excelled with a smaller network. Additionally, adjusting learning rates showed that while a default rate favored off-policy algorithms, a lower-than-default learning rate enhanced PPO’s performance, as demonstrated in Fig. 3.

Success rates of fine-tuned algorithms are shown in Fig. 2(b). Overall, SAC’s success rate increased from 66% to 70% and PPO’s from 70% to 84%, highlighting the efficacy of hyperparameter tuning. Conversely, TD3 and DDPG maintained stable success rates, since the default settings were optimal within the hyperparameter ranges investigated in this work.

### Tuned hyperparameters in DualDeviceNav environment

Success rates observed during training in DualDeviceNav, with hyperparameters tuned in ArchVariety, are shown in Fig. 2(c). Among the algorithms evaluated, TD3 demonstrated the best performance, achieving a peak success rate of 68% with an average procedure time of 214.05 s. In contrast, despite DDPG showing significantly lower efficacy, with a success rate of 24%, it navigated with the shortest procedure time of 88.62 s. Conversely, PPO had the longest procedure time of 574.33 s.

**Table 1:** Results of experiments comparing RL algorithms in ArchVariety and DualDeviceNav

Experiment	Algorithm	Success rate (%)	Procedure time (s)	Path ratio (%)	Exploration steps
ArchVariety (default)	DDPG	80	6.87	78.8	6.75 $\times$ 10$^6$
	TD3	79	14.04	61.1	8.25 $\times$ 10$^6$
	SAC	66	7.49	71.0	7.25 $\times$ 10$^6$
	PPO	70	5.5	67.7	7.5 $\times$ 10$^6$
ArchVariety (tuned)	SAC	70	7.72	71.6	6.75 $\times$ 10$^6$
	PPO	84	5.08	77.3	9.75 $\times$ 10$^6$
DualDeviceNav (tuned)	DDPG	24	88.62	55	1.0 $\times$ 10$^6$
	TD3	68	214.05	62	2.5 $\times$ 10$^6$
	SAC	58	182.98	66	8.25 $\times$ 10$^6$
	PPO	41	574.33	66	6.5 $\times$ 10$^6$

## Discussion

### Comparison of algorithms with default hyperparameters

The learning curves in Fig. 2(a) show that DDPG outperformed other algorithms in terms of initial learning speed and maintaining a high success rate. However, DDPG exhibited significant fluctuations, suggesting instability during training, likely due to the overestimation of Q-values. In contrast, TD3 showed more stable performance, avoiding the dips seen with DDPG. This stability can be attributed to TD3’s dual Q-function architecture, which mitigates the overestimation issue of DDPG. SAC demonstrates a slower initial learning phase but maintains a relatively stable performance throughout training, with fewer fluctuations compared to DDPG and PPO. While its peak success rate is lower than that of DDPG and TD3, SAC’s emphasis on balancing exploration and exploitation ensures consistent and reliable performance over time. PPO, on the other hand, showed a rapid initial increase in success rate, comparable to DDPG and TD3, but later exhibited significant instability. This instability is likely due to the on-policy nature of PPO, as it only uses data collected from the most recent policy to train at each timestep. As a result, a series of suboptimal actions can lead to fluctuations between non-optimal paths, preventing consistent convergence toward an optimal solution. This comparison underscores the strengths of off-policy algorithms in terms of sample efficiency and stability, as these algorithms can reuse past experiences. This advantage is evident in their ability to achieve and maintain higher success rates with less fluctuation than PPO.

### Network size and learning rate adjustments

Results from varying network sizes (Fig. 3) indicate that larger networks lead to higher success rates in off-policy algorithms. This suggests that larger networks are more adept at capturing the essential features needed for effective performance in this RL task. For PPO, the superiority of the smaller network might suggest that the larger network, despite its higher capacity, could be overfitting to specific states or rewards in the environment. Overfitting in larger networks can result in poorer generalization to new or varied states encountered during exploration. These results indicate that SAC and PPO are sensitive to network size. In contrast, DDPG and TD3 show less sensitivity to network size, with both large and small networks yielding similar results.

In terms of learning rate, the off-policy algorithms achieved higher success rates at their default settings, while PPO reached its highest success rate with a lower-than-default value but displayed instability. For PPO, increasing the learning rate improved stability but reduced success rates. DDPG performed best when actor and critic learning rates were equal. Increasing the critic’s rate beyond the actor’s destabilized training, leading to poorer policy learning and overall performance. Similarly, an overly aggressive learning rate, as seen with SAC, significantly hindered performance.

Among the four algorithms evaluated, PPO exhibited the greatest sensitivity to the tested hyperparameters. This sensitivity is attributed to its on-policy nature, where only data collected from the current policy are used for training at each timestep, making it inherently less stable compared to the off-policy algorithms. Moreover, the outcomes of this hyperparameter tuning experiment underscore the critical impact of hyperparameter selection on an algorithms’s final performance.

### Comparison of algorithms within the DualDevice environment

The results show that TD3 achieved the highest success rate among the algorithms (see Fig. 2(c)), with the caveat that it exhibited considerable variability and instability during training. In contrast, SAC demonstrated more gradual and stable updates, though the noise observed in its results suggests challenges in maintaining a balance between exploration and efficient learning. PPO exhibited substantial instability, again due to the inherent limitations of on-policy learning. Meanwhile, DDPG showed the poorest performance, with consistently low success rates throughout the exploration steps, indicating a struggle to learn effectively in this environment.

The observed differences in algorithm performance are likely due to hyperparameters, which were optimized for a simpler environment. These settings did not generalize well to the more complex scenario encountered, characterized by longer navigation paths and simultaneous use of two instruments. This underscores the importance of fine-tuning hyperparameters to enhance performance as task complexity increases.

In a related study, Robertshaw et al. achieved a success rate of 96% with a procedure time of 24.9 s, using the same reward function and environment as this study [21]. However, they employed a custom SAC implementation featuring a long short-term memory (LSTM)-based observation embedder that learns trajectory-dependent states. This was specifically tuned to the task through numerous iterations, which likely contributed to their superior performance compared to the SB3-based feedforward architecture. Without this benchmarking, comparing differences in algorithm implementations and assessing their relative effectiveness would have been challenging.

### Limitations and future Work

This study provides *in silico* benchmarks, enabling related work to compare algorithms and drive innovation by easily identifying the best-performing algorithm. However, *in vitro* experiments are essential for effective clinical translation, as they evaluate algorithms in realistic conditions to ensure adaptability to real-world settings. Future work will integrate physical models to validate these algorithms in practical scenarios.

Relying on a single aortic arch type for ArchVariety, a single mesh for DualDeviceNav, and the sTEVE simulation platform limits the generalizability of findings to diverse anatomical variations and simulation platforms. Expanding *in silico* validation to include a broader range of anatomies and platforms (e.g., CathSim) will improve the effectiveness of future *in vitro* experiments. Additionally, while this study relied on tracking coordinates of the instrument tip in simulations, a similarly clinically relevant approach could include image-based tracking which leverages the entire fluoroscopic imaging, rather than the tips of the devices used in our method.

Although this study highlights the importance of tuning network size and learning rate, it lacked a comprehensive hyperparameter search and systematic random seed exploration. Moreover, in the DualDeviceNav environment, the same hyperparameters tuned for the ArchVariety environment were applied to assess their generalizability. While this approach provided insights into algorithm robustness, environment-specific tuning could further improve performance. Future work will include a more thorough hyperparameter search, exploring parameters such as batch size and network depth, alongside environment-specific tuning and random seed exploration to enhance robustness and optimize performance.

The singular reward function may not suit all RL algorithms equally; therefore, future work should explore alternative formulations tailored to different algorithms. A further limitation of our work is the exclusive focus on RL algorithms, without considering alternative approaches such as imitation learning or model-based methods. Incorporating these approaches into future iterations of the benchmark would enable a broader evaluation of diverse learning strategies.

## Conclusion

Benchmarking is crucial for advancing autonomous solutions in endovascular procedures, as it offers a framework for comparing the effectiveness of different algorithms. RL algorithms from the SB3 library have been successfully evaluated on endovascular benchmark environments from stEVE. The results demonstrate that PPO achieved the highest maximum success rate of 84% and the shortest procedure time of 5.08 s in ArchVariety, while TD3 led in DualDeviceNav with a success rate of 68% and a procedure time of 214.05 s. This work lays the foundation for future research to compare RL algorithms, helping to identify the most effective algorithm for the presented tasks and highlighting areas for improvement among the RL algorithms tested.

## Supplementary Information

Below is the link to the electronic supplementary material.

Supplementary file 1 (pdf 357 KB)

Supplementary file 2 (mp4 35040 KB)

## Declarations

### Code availability

The code is available at https://github.com/Farhana-M/RL-ENDOVASCULAR
