# Supplementary material for: Benchmarking reinforcement learning algorithms for autonomous mechanical thrombectomy
Source: Int J Comput Assist Radiol Surg. 2025 Apr 29;20(6):1231–8. doi: 10.1007/s11548-025-03360-x (PMC12167280; doi:10.1007/s11548-025-03360-x)
Supplement: Supplementary file 1 — (pdf 357 KB) [file 11548_2025_3360_MOESM1_ESM.pdf]

Supplementary material: Benchmarking Reinforcement Learning Algorithms for Autonomous Mechanical Thrombectomy

Farhana Moosa[1†], Harry Robertshaw[1†], Lennart Karstensen[2], Thomas C Booth[1,3], Alejandro Granados[1*]

[1]School of Biomedical Engineering and Imaging Sciences, Kings College London, United Kingdom.
[2]AIBE, Friedrich-Alexander University Erlangen-Nürnberg, Germany.
[3]Department of Neuroradiology, Kings College Hospital, United Kingdom.

*Corresponding author(s). E-mail(s): alejandro.granados@kcl.ac.uk;
[†]These authors contributed equally to this work.

Table S1 Default hyperparameters for DDPG, TD3, PPO and SAC in Stable Baselines3 (SB3)

**Hyperparameter**	**DDPG**	**TD3**	**SAC**	**PPO**
Network architecture	[400,300]	[400,300]	[256,256]	[64,64]
Activation	ReLU	ReLU	ReLU	ReLU
Optimizer	Adam	Adam	Adam	Adam
Learning rate	0.001	0.001	0.0003	0.0003
Target update rate	1 episode	1 episode	1 episode	2048 steps
Batch size	256	256	256	64
Epochs	-	-	-	10
Discount factor (γ)	0.99	0.99	0.99	0.99
Replay Buffer Size (R)	1,000,000	1,000,000	1,000,000	-
Clip range (ϵ)	-	-	-	0.2
Generalized advantage estimation (λ)	-	-	-	0.95
Soft update coefficient (τ)	0.005	0.005	0.005	-
Entropy coefficient (α)	-	-	auto	0
vf coefficient	-	-	-	0.5
Action noise ($\mathcal{N}$)	None	None	None	-
Policy delay	-	2	-	-
Learning starts	100	100	1000	-
Train frequency	1	1	1	-
Gradient steps	1	1	1	-
Max gradient norm	-	-	-	0.5
Normalize advantage	-	-	-	True

Table S2 Hyperparameter search space for actor and critic learning rates

**Algorithm**	**Actor Learning Rate Search Space**	**Critic Learning Rate Values Tested**	**Additional Details**
DDPG	Logarithmic, 1×10^{-5} to 1×10^{-2} (25 intervals)	1×10^{-4}, 1×10^{-3}, 1×10^{-2}	Actor learning rates tested were 1×10^{-5}, 1×10^{-4}, and 1×10^{-3} for these critic learning rates.
TD3	Logarithmic, 1×10^{-5} to 1×10^{-2} (25 intervals)	1×10^{-4}, 1×10^{-3}, 1×10^{-2}	Same as above.
SAC	Logarithmic, 1×10^{-5} to 1×10^{-2} (25 intervals)	1×10^{-4}, 1×10^{-3}, 1×10^{-2}	Same as above.
PPO	Logarithmic, 1×10^{-5} to 1×10^{-2} (25 intervals)	Not applicable	

Note: For the preliminary grid search, navigation tasks were performed for 250,000 exploration steps. Evaluations were conducted every 25,000 steps for 25 episodes. Key metrics recorded at each evaluation step included success rate, procedural time, and path ratio.

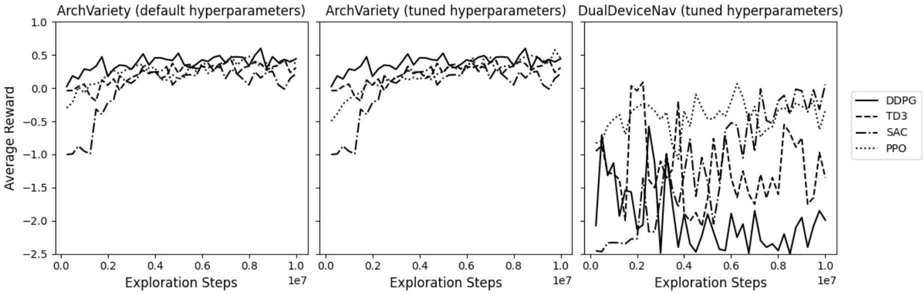

Fig. S1 Training curves showing the average reward across exploration steps for ArchVariety under default (left) and tuned (middle) hyperparameters, as well as DualDeviceNav under tuned hyperparameters (right) for DDPG, TD3, SAC, and PPO algorithms.

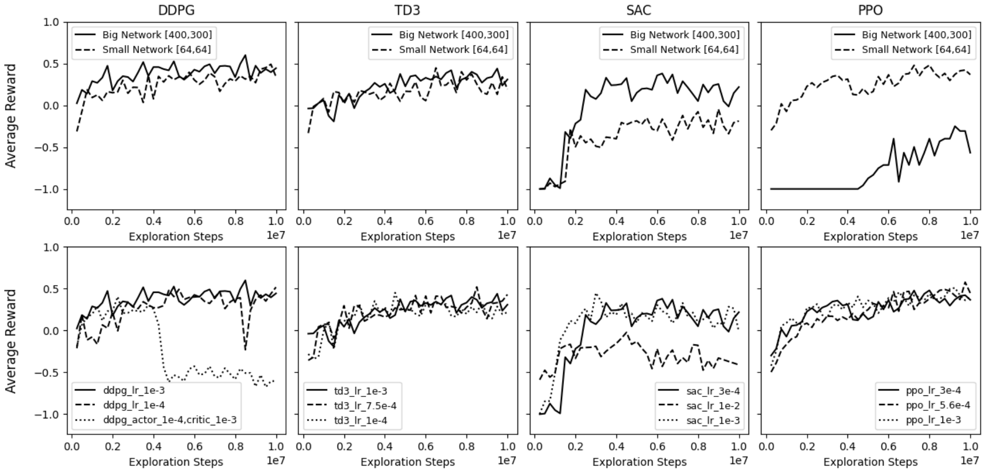

Fig. S2 Top row: Training curves depicting the average reward across exploration steps for big and small networks for DDPG, TD3, SAC, and PPO algorithms. Bottom row: Training curves showing the effect of three distinct learning rates on the average reward for each algorithm.
